# Persistence of Abscisic Acid Analogs in Plants: Chemical Control of Plant Growth and Physiology

**DOI:** 10.3390/genes14051078

**Published:** 2023-05-13

**Authors:** Christine H. Nguyen, Dawei Yan, Eiji Nambara

**Affiliations:** Department of Cell & Systems Biology, University of Toronto, 25 Willcocks St., Toronto, ON M5S 3B2, Canada

**Keywords:** abscisic acid, ABA analog, ABA 8′-hydroxylase, cis–trans isomerization, glycosylation, persistence, plant growth regulator (PGR), reactive oxygen species (ROS)

## Abstract

Abscisic acid (ABA) is a plant hormone that regulates numerous plant processes, including plant growth, development, and stress physiology. ABA plays an important role in enhancing plant stress tolerance. This involves the ABA-mediated control of gene expression to increase antioxidant activities for scavenging reactive oxygen species (ROS). ABA is a fragile molecule that is rapidly isomerized by ultraviolet (UV) light and catabolized in plants. This makes it challenging to apply as a plant growth substance. ABA analogs are synthetic derivatives of ABA that alter ABA’s functions to modulate plant growth and stress physiology. Modifying functional group(s) in ABA analogs alters the potency, selectivity to receptors, and mode of action (i.e., either agonists or antagonists). Despite current advances in developing ABA analogs with high affinity to ABA receptors, it remains under investigation for its persistence in plants. The persistence of ABA analogs depends on their tolerance to catabolic and xenobiotic enzymes and light. Accumulated studies have demonstrated that the persistence of ABA analogs impacts the potency of its effect in plants. Thus, evaluating the persistence of these chemicals is a possible scheme for a better prediction of their functionality and potency in plants. Moreover, optimizing chemical administration protocols and biochemical characterization is also critical in validating the function of chemicals. Lastly, the development of chemical and genetic controls is required to acquire the stress tolerance of plants for multiple different uses.

## 1. Introduction

### Introduction to ABA

Plants experience frequent changes in their environment. They possess the ability to sense, respond, and adapt to their surroundings to produce seeds in a given place [1]. Plant hormones are responsible for adapting plants to environmental stimuli. Abscisic acid (ABA) is one of the five classic phytohormones responsible for regulating plant growth and physiology, including responses to abiotic and biotic stresses and seed dormancy and germination [2].

ABA is a sesquiterpene produced from the cleavage of C40 carotenoids [3]. In plants, ABA levels must be tightly controlled and regulated by biosynthetic and inactivation pathways. ABA is inactivated by two types of catabolic pathways, oxidation and conjugation [4]. The oxidation of ABA results in its hydroxylation at methyl groups (C-7′, C-8′, and C-9′) [3]. ABA 8′-hydroxylation forms 8′-hydroxy ABA, which quickly isomerizes to phaseic acid (PA). PA is further catalyzed to dihydrophaseic acid (DPA) [3]. The CYP707A subfamily of cytochrome P450 monooxygenases catalyzes the 8′-hydroxylation of the ABA [5,6]. In response to drought stress and in seeds, *cyp707a* mutants accumulate higher levels of ABA than wild type [7,8]. For conjugation, ABA is bound to glucose to generate ABA glucosyl ester (ABA-GE). This step is catalyzed by uridine diphosphate glucosyltransferases (UGTs), a group of plant glucosyltransferase superfamily [3]. Over-expression of an ABA glycosyltransferase increases ABA-GE levels but has an insignificant effect on ABA levels [9]. The conversion between ABA and ABA-GE is a reversible process, and the hydrolysis of ABA-GE is regulated by glucosidases, including β-glucosidase homolog1 (BG1) and BG2 [10,11]. ABA catabolic enzymes are responsible for decreasing ABA levels when the level has to be low.

**Figure 1:**
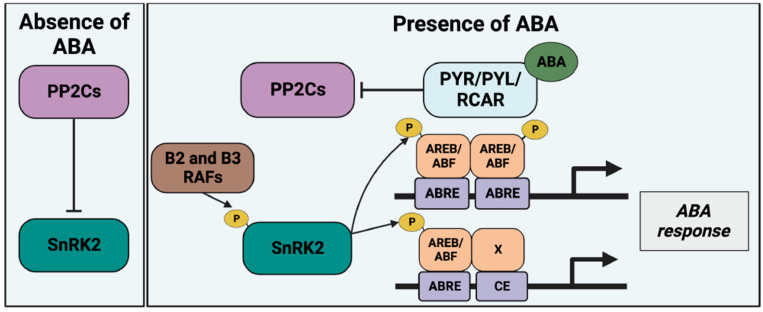
ABA signaling and ABA-mediated transcription. Left column, SnRK2 kinases are inactivated by clade A PP2Cs in the absence of ABA. Right column, The ABA-bound PYR/PYL/RCAR receptors inhibit the PP2C activity. B2/B3 Raf kinases phosphorylate SnRK2s [25]. ABRE/ABF transcription factors are phosphorylated by derepressed SnRK2 and trigger ABA-responsive element (ABRE)-mediated transcription. ABRE-mediated transcription includes the multi-copy ABRE in the promoter or one-copy ABRE with the coupling elements (CE). Several distinct CEs are reported with corresponding transcription factors, indicated by X. Figure created with BioRender (https://biorender.com/, accessed on 6 March 2023).

The core components of the ABA signaling pathway include the family of pyrabactin resistance 1/PYR1-like/regulatory components of ABA receptors (PYR/PYL/RCAR), clade A type 2C protein phosphatases (PP2C-As), and Sucrose non-fermenting1 (SNF1)-related protein kinase 2 (SnRK2) kinases [2]. The binding of ABA to the PYR/PYL/RCAR receptors triggers the ABA signaling by repressing the activity of PP2C-As [12,13]. PP2C-As are negative regulators of ABA signaling and can dephosphorylate SnRK2s in the activation loop to maintain SnRK2 inactive [14,15,16,17]. ABA binds to PYR/PYL/RCAR receptors and triggers a change in the configuration of the receptor through the gate–latch–lock mechanism, allowing PYR/PYL/RCAR proteins to bind to and inhibit PP2C-As [18,19,20]. This interaction restores the phosphorylation of the SnRK2s activation loop to activate downstream targets, including ABA-responsive elements (ABRE)-binding factors (ABF) transcription factors and ion channels responsible for stomatal closure [17,21,22,23,24] (Figure 1).

There are fourteen members of the PYR/PYL/RCAR receptors in Arabidopsis, and they can be distinguished based on their oligomeric states in the absence of ABA [26,27,28]. PYR1, PYL1, and PYL2 belong to the homodimeric receptors, PYLs 4-12 are monomeric, and PYL3 is in monomer–dimer exchange [26,27]. The equilibria between the monomers and dimers of the PYR/PYL/RCAR members are correlated with their ABA-binding affinities [26]. It has been shown that dimeric receptors have a lower intrinsic affinity for ABA and must dissociate into monomers before interacting with and inhibiting PP2C-As [26,27]. Therefore, monomeric receptors have a competitive advantage for ABA binding compared to dimeric receptors [26,27].

The primary response of ABA-mediated transcription involves the SnRK2 phosphorylation of ABRE-binding proteins (AREB)/ABF transcription factors [22] (Figure 1). AREB/ABF belong to the subclass of basic domain leucine zipper (bZIP) transcription factors that bind to the ABRE in the promoters of ABA-regulated genes [29]. A full induction of ABRE-mediated transcription requires multi-copies of ABREs [30]. Otherwise, one-copy ABRE requires the proximally located coupling elements (CEs) to induce ABA- and ABRE-mediated transcription [30] (Figure 1). The single-copy ABRE-containing genes count over 50% of the ABA-regulated genes [31]. This suggests that AREB/ABF-mediated transcription involves interactions with other transcription factors. This might make the ABA-regulated transcriptome more diverse. This primary transcriptional response regulates the expression of other transcription factors, such as the homeodomain–leucine zipper (HD-ZIP) proteins and NAC (NAM, ATAF1/2, and CUC2) family proteins [29,32,33,34]. These transcription factors induce secondary transcriptional responses. Transcriptome analyses have indicated that there are thousands of genes that are regulated by ABA application in various plants [35].

ABA-regulated transcriptomes cover diverse functional categories, such as signaling, transport, transcription, and metabolism [2,36,37]. This is consistent with the notion that ABA-induced stress tolerance involves multiple mechanisms in different biological processes. For example, ABA-regulated genes include those related to reactive oxygen species (ROS) homeostasis [38]. The proper levels of ROS are required for plant growth, development, and stress response. Thus, both low and high ROS levels cause a negative impact on plant performance. Hydrogen peroxide (H_2_O_2_) produced by NADPH oxidases acts as a secondary signal to promote ABA-mediated stomatal closure [39]. Proper ROS levels are required for seed germination and to break dormancy [40]. In contrast, high levels of ROS induced by environmental stresses cause oxidative damage in plants. ABA induces the expression of genes for ROS detoxification, such as catalases, superoxide dismutases, and peroxidases, to scavenge excess ROS [38]. It is known that antioxidant enzyme levels are correlated with plant stress tolerance [41]. Therefore, antioxidant activity is an important criterion for evaluating plant stress tolerance.

## 2. ABA and Agriculture

### 2.1. ABA as a Plant Growth Regulator

Mitigating environmental stress is essential for crop productivity. Chemical modulation of ABA signaling is an effective strategy for agricultural practices to avoid unwanted crop damage from various stresses. However, the labile nature of ABA is a bottleneck to its agricultural uses. The persistence of applied ABA in plants depends on multiple factors, such as photostability and intrinsic catabolic pathways. The altered persistence of ABA agonists is most readily achieved with structural analogs because their metabolic fates are more predictable than those of other ABA agonists identified by high-throughput screening against combinatorial chemical libraries.

### 2.2. Registered Uses of (S)-ABA in Agriculture

**Figure 2:**
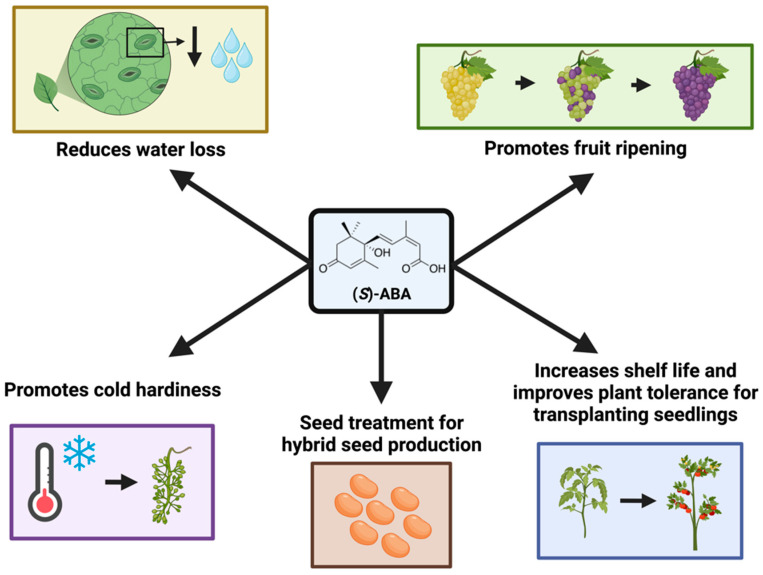
Registered uses of (*S*)-ABA in agriculture. ABA has been registered for uses in anti-transpiration, promoting fruit ripening, cold hardiness, hybrid seed production, and acquiring plant tolerance to transplanting. Figure created with BioRender (https://biorender.com/, accessed on 6 March 2023).

(*S*)-ABA has been registered for different uses in agriculture (Figure 2) [42,43,44]. A well-known function of ABA is to improve plant tolerance to abiotic stresses, such as drought and cold. (*S*)-ABA application is used for improving plant tolerance to transplanting shock for seedlings [43,45], as well as cold tolerance [46].

ABA controls fruit ripening [47]. Aside from the prominent roles of ethylene in climacteric fruit ripening, ABA promotes the ripening of non-climacteric fruits [48]. Its action is often independent but it is also interactive with other hormones, such as ethylene and auxin [48]. These hormones induce fruit ripening processes, including the induction of genes related to cell wall loosening enzymes for fruit softening and flavonoid biosynthesis for fruit coloration. ABA is also effective in promoting fruit set in apples [49].

## 3. ABA Analogs

Hundreds of ABA structural analogs (ABA analogs) have been reported [50,51,52]. ABA analogs are synthetic chemicals in which functional groups of ABA are modified [51]. In this article, we distinguish ABA analogs from the functional ABA mimics that are selected as ABA agonists; those structures are distinct from the backbone of ABA. Early works evaluated whether ABA analogs induce (i.e., as agonists) or inhibit (i.e., as antagonists) ABA-like activities in bioassays, such as inhibition of growth and germination. Thus, these bioassays do not distinguish whether these analogs are bioactive forms or acting as precursors. Nonetheless, structure–activity relationship studies have contributed remarkably to understanding the functionality of each functional group of ABA [51]. The identification of ABA receptors allows the in vitro evaluation of ABA analogs by the receptor-mediated inhibition of PP2C-A activities [50]. These analyses have revealed that most of the structural features of ABA are required for it to function as a short-lived specific signal molecule. Reported functions of ABA analogs include ABA agonists and antagonists and inhibitors of ABA catabolism [53,54]. Additionally, the improved resolution of ABA receptor structures allows high-throughput screening in vitro for functional ABA mimics with distinct backbones from (+)-ABA from random chemical structures [55].

The development of long-lasting ABA agonists is a challenge. Indeed, a number of functional ABA mimics act as selective agonists to dimeric receptors, but their effects on monomeric receptors are often weak. It should be designed under structural restrictions to maintain high affinity and specific binding to ABA receptors, preferentially all ABA receptors (i.e., pan-receptor agonists) from various plant species. Recent structural studies on the PYR1 ABA receptor reveal that 21 amino acid residues in the binding pocket are involved in stabilizing ABA binding [53]. These functional groups are in good agreement with the prediction from the structure–activity relationship study using bioassays. Modifications of the structure of ABA also influence the binding to its catabolic enzymes, which in turn alters the persistence of ABA analogs [56]. The persistence of these chemicals in plants impacts the potency of these chemicals as plant growth regulators. In this review, we update the current understanding of the persistence of ABA analogs.

## 4. Persistence of ABA Analogs

The persistence of applied chemicals is a critical factor for its efficacy [57]. Extensive efforts have been made to develop long-lasting ABA analogs in plants.

### 4.1. Photostable ABA Analogs

**Figure 3:**
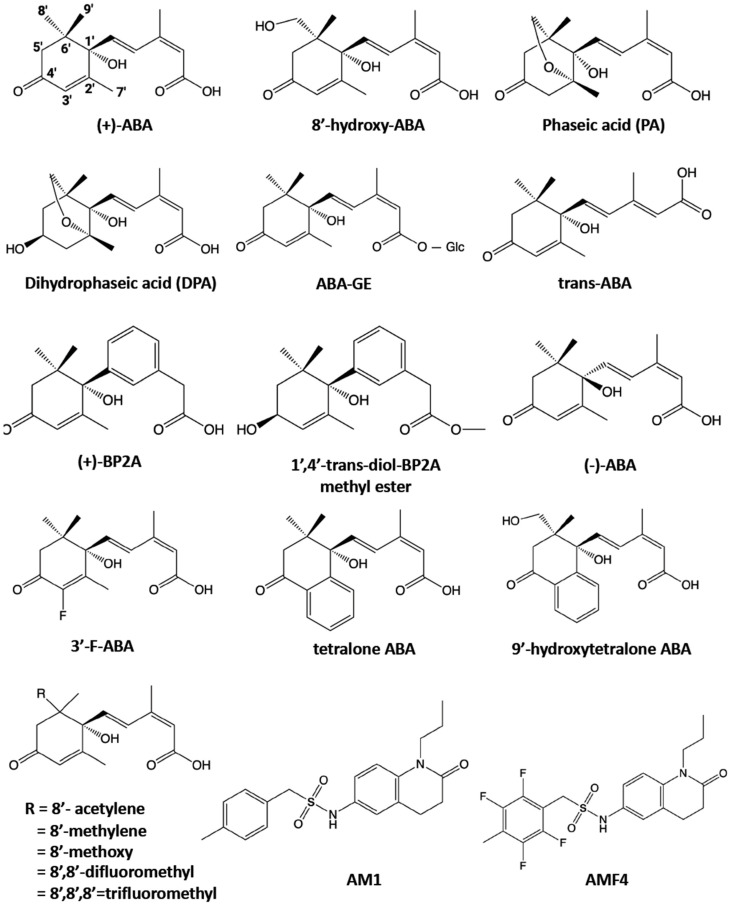
ABA-related compounds introduced in this article. (+)-ABA is depicted with numbering for ring carbons in position. 8′-hydroxy-ABA, phaseic acid (PA), dihydrophaseic acid (DPA), ABA glucose ester (ABA-GE), and *trans*-ABA are endogenous metabolites of ABA. ABA mimic 1 (AM1) and AM1 fluorine 4 (AMF4) are the functional mimics of ABA, while others are ABA analogs.

ABA is a photosensitive chemical. The dienoic acid side chain and the cyclohexenone ring of ABA are sensitive to irradiation with mild ultraviolet (UV) (365 nm) and strong UV-B (280–320 nm), respectively [58]. Light irradiation results in the cis–trans isomerization of the side chain of (+)-ABA from a 2*Z* (*cis*) configuration to biologically inactive 2*E*-*trans*-ABA [59] (Figure 3). The cis–trans isomerization of the ABA side chain is reversible and maintains a given ratio in equilibrium [60].

Much effort has been made to modify the side chain of ABA to prevent photolability. (+)-BP2A was designed to alter the side chain while maintaining the requirement for binding to PYR/PYL/RCAR receptors [58] (Figure 3). (+)-BP2A displays enhanced photostability under sunlight and UV-B irradiation [58]. (+)-BP2A acts as the pan-ABA agonist that mediates the PYL-mediated PP2C inhibition with all receptor types. Moreover, methyl 1′,4′-trans-diol-BP2A, a (+)-BP2A derivative that reduced the C-4′ ketone in the ring structure, shows strong tolerance to UV-B-induced photolysis. However, it merely shows the PYL5-specific agonist activity in the PYL-mediated PP2C inhibition assay, but it does not interact with the other nine PYL receptors tested. Interestingly, despite its weak activity in vitro, methyl ester of 1′,4′-trans-diol-BP2A showed more potent biological activities than (+)-BP2A in some in vivo assays, such as the inhibition of seed germination in Arabidopsis and tomato [58] (Figure 3). The metabolite fate experiment demonstrates that it is converted to (+)-BP2A in plants, thus acting as a precursor of (+)-BP2A. Hence, this could be a powerful photostable ABA agonist in the field under sunlight.

### 4.2. Analogs Resistance to ABA 8′-Hydroxylase

(−)-ABA is a classic example of a long-lasting ABA analog. Early chemical syntheses of ABA produced the racemic mixture of natural (+)-ABA and non-natural (−)-ABA. (−)-ABA possesses ABA-like activities, although its efficacy varies depending on bioassays in various plant species. In vitro PP2C inhibition assays show that the affinity of (−)-ABA to all Arabidopsis ABA receptors is very weak compared to (+)-ABA [61,62]. Instead, (−)-ABA is not a substrate of ABA 8′-hydroxylase [5]. Thus, (−)-ABA is more persistent than (+)-ABA in Arabidopsis plants [63,64]. Interestingly, in *Marsilea quadrifolia*, an aquatic plant, application of (+)-ABA induces a petiole elongation characteristic to the aerial growth, called heterophylly. Application of (−)-ABA induces heterophylly more effectively than (+)-ABA [63]. Feeding studies with deuterated compounds showed that the enhanced bioactivity of (−)-ABA is due to both persistence and an increase in endogenous ABA levels [63]. This also suggests that increasing endogenous ABA levels is a potential mechanism of ABA analogs as plant growth substances to confer ABA-like bioactivity.

Modification of the C-8′ methyl group is effective in developing ABA analogs resistant to CYP707As (Figure 3). Modifying C-8′ and C-9′ with small functional groups has negligible effects on the binding to ABA receptors, but these modifications impact the persistence of these analogs [62]. For example, 8′,8′,8′-trideuterato ABA is long-lasting in plants [65,66]. Because the C-D bond is stronger than C-H, the deuterated methyl group at C-8’ is more tolerant than the methyl group to oxidation. 8′-acetylene ABA is resistant to ABA 8′-hydroxylase. Thus, it persists for longer periods in plants [67]. 8′-Methylene ABA is slowly metabolized to epoxides and displays greater potency for the inhibition of the germination of cress and wheat and decreased transpiration of wheat seedlings [68]. Additionally, 8′-methoxy ABA, 8′,8′-difluoro- and 8′,8′,8′-trifluoro ABA and 5′α,8′-cycloABA also act as long-lasting analogs displaying potent biological activities [69,70,71]. Some of these analogs act as inhibitors of ABA 8′-hydroxylases [56,67]. Thus, the effect of these analogs on plant stress tolerance is attributed to both long-lasting agonists and the increase in endogenous ABA levels by inhibiting ABA 8′-hydroxylases.

### 4.3. ABA Analogs Resistant to Intramolecular Cyclization after C-8′ Hydroxylation

ABA 8′-hydroxylation is the committed step in a major ABA catabolic pathway. The inactivation of ABA occurs gradually in a stepwise process to form the complete inactive DPA. Eight’-hydroxy ABA itself still possesses substantial ABA-like activity [72,73,74] (Figure 3). It is efficiently isomerized to PA, which is a less active metabolite in an equilibrium reaction [75]. ABA analogs with decreased conversion to the PA-like bicyclic form persist in biologically active hydroxy ABA and prevent further reduction to form fully inactive DPA-like molecules.

The equilibrium between the 8′-hydroxylated form and the PA-like bicyclic form is disturbed in 3′-fluoro ABA (3′-F-ABA) (Figure 3) [72]. Concomitant with the decreased conversion to PA-like bicyclic form, 3′-fluoro-8′-hydroxy ABA (3′-F-8′-hydroxy ABA) is more stable and long-lasting than 8′-hydroxy ABA in rice cell culture [72]. Although weaker than 3′-F-ABA, 3′-F-8′-hydroxy ABA possesses significant bioactivity compared to 3′-fluoro PA (3′-F-PA) in various bioassays [72]. The authors discussed that the long-lasting nature of 3′-F-8′-hydroxy ABA could contribute to the bioactivity of 3′-F-ABA in long-term assays [72].

Tetralone ABA is a bicyclic ABA analog with a benzene ring fused to the C-2′ and C-3′ positions of ABA (Figure 3) [76]. The hydroxylated analog (9’-hydroxytetralone ABA; Figure 3) cannot proceed to the intramolecular isomerization to form the inactive PA-like structure [76]. Therefore, blocking cyclization stabilizes hydroxytetralone ABA in plants, similar to 3′-F-8′-hydroxy ABA. Tetralone ABA is a pan-receptor agonist that displays high affinity to all types of ABA receptors from Arabidopsis and wheat equivalent to (+)-ABA in vitro [77], indicating that the addition of a benzene ring does not affect the affinity to ABA receptors. Interestingly, its bioactivity is more pronounced than (+)-ABA in various bioassays in vivo [78]. Hydroxylated tetralone ABA (equivalent to 8′-hydroxy ABA) possesses ABA-like activities and is much more potent than PA in various bioassays in Arabidopsis [78]. The potency of hydroxy tetralone ABA varies among bioassays. Consistently, in vitro PP2C inhibition assay with ABA receptor showed that the hydroxylated tetralone ABA varies its affinity among the members of ABA receptors, serving as a selective ABA agonist [78]. Nonetheless, the tetralone ABA’s affinity to ABA receptors is equivalent to (+)-ABA [77]. Thus, it is likely that the persistence of hydroxylated tetralone ABA might contribute to the observed increase in the bioactivities of tetralone ABA. It is also worth noting that the modifications of the benzene ring in tetralone ABA can confer ABA antagonist activities [79].

### 4.4. ABA Analogs and Functional Mimic Resistant to UGT and Xenobiotic Metabolism

Plants are constantly exposed to environmental chemicals. Some of them could be toxic when accumulated to a certain level. Thus, plants require xenobiotic metabolism enzymes to detoxify and eliminate these xenobiotics. These enzymes catalyze reactions for the modification and conjugation of xenobiotics to small molecules (such as sugars and glutathione) prior to the transport. Xenobiotic metabolism requires multiple enzymes, such as cytochromes P450, glutathione S-transferases, and UGTs, for limited absorption, increased detoxification, and facilitated elimination, thus reducing the level of the toxic compound [80].

Applied ABA at high concentrations in plants is quickly converted to sugar conjugate forms [81]. This presumably resembles xenobiotic metabolism, although some plant UGTs are selective to ABA. Glycosylation of ABA analogs was characterized for Arabidopsis UGT71B6 [82]. UGT71B6 is an ABA glycosyltransferase (ABA UGT) with the highest selectivity to (+)-ABA among 6 ABA UGTs identified from a systematic screening for Arabidopsis UGTs [83]. For example, (-)-ABA is a poor substrate for UGT71B6 [82]. Interestingly, 8′-acetylene ABA is not a substrate of UGT71B6 in vitro, while tetralone ABA is a three-times better substrate than (+)-ABA in vitro [9]. The in vitro substrate preference of UGTs is well correlated with seedling growth inhibition in UGT71B6 overexpressors of Arabidopsis treated with these ABA analogs [9]. In addition, UGT71C5 is also reported to be a crucial ABA UGT for ABA homeostasis in Arabidopsis [84]. It would be important to analyze the persistence of these ABA analogs in plants because multiple selective and promiscuous enzymes are involved in ABA glycosylation.

ABA mimic 1 (AM1) fluorine derivatives (AMFs) are designed from AM1 or quinabactin as a lead compound by adding fluorine atoms to the methylbenzene ring [85] (Figure 3). AM1/quinabactin is a dihydroquinolinone–sulfonamide that was selected as a potent ABA agonist [86]. Among AMF derivatives, AMF4 showed more prolonged ABA-like activities than ABA in plants [85]. AMF4 possesses two features to gain the potency for anti-transpiration. One is enhanced affinity to the ABA receptor, and the other is prolonged persistence in plants [85]. The other is that the persistence of AMF4 is perhaps due to the resistance to physiological oxidation, which often triggers xenobiotic metabolism. The stronger binding of C-F than C-H supports this notion.

## 5. Conclusions and Perspectives

ABA is an important stress signal that acquires stress tolerance in plants. Therefore, genetic and chemical approaches to modulate ABA functions have been extensively investigated. This review focuses on the chemical approach, especially the persistence of ABA analogs. Another review article has summarized the genetic and chemical approaches using ABA mimic to modify ABA metabolism and signaling genes [87].

The increase in the yield and survival rate of plants subjected to different stressors is the most important criterion to evaluate how analogs effectively stimulate ABA signaling and enhance plant stress tolerance. In addition, it is also important to characterize the mode of the analogs’ action. Even though ABA analogs are expected to mimic ABA, the metabolism, localization, and persistence of these analogs is not the same. Therefore, the same potency between two analogs does not necessarily mean that both analogs act in the same mode of action. To overcome this issue, it would be worth evaluating the metabolic fate and persistence of these analogs, as highlighted in this article. Another option is to perform biochemical analyses on plants treated with these analogs, such as measuring antioxidant activity. Because ABA action is systemic, analytical methods that evaluating individual organs would be advantageous to understand the mode of the analogs’ action.

Long-lasting ABA analogs have been well investigated for ABA agonists. The same scheme can be applicable to enhance the potency of ABA antagonists. ABA antagonists promote seed germination and growth promotion under inappropriate conditions [88,89]. An ABA antagonist is also registered for use to improve pathogen resistance [90]. It would be worth carrying out an investigation of persistence to develop potent ABA antagonists. In particular, some ABA antagonists include tetralone ABA derivatives [79]. It is possible that some of these antagonists have defects in intramolecular cyclization to form a PA-like structure. It would be interesting to examine the persistence of these compounds for a better understanding of the mode of action.

The impact of the persistence of ABA analogs in plants might depend on the assay systems, such as how and when these analogs are applied to plants. For example, the impact of persistence might be masked when plants are continuously exposed to these chemicals, such as root growth and germination assays. Plant ages also potentially affect the impact of chemical persistence. The activities of catabolism and xenobiotic metabolism vary depending on the plant developmental stages. Therefore, regarding the development of agrochemicals, it is ideal to evaluate their potency in assays similar to their uses.

The persistence of ABA analogs has a significant impact on the potency of these chemicals in vivo. A recent report showed that tetralone ABA recovers the growth retardation of the Arabidopsis ABA-deficient *aba2* mutant 100 times more effectively than ABA in a one-time application [91]. This is consistent with the hypothesis that long-lasting ABA analogs are more potent when the amount of application is restricted. The decrease in chemical applications impacts environmentally friendly and cost-effective practices in agriculture. Importantly, it is difficult to predict the best timing for applying agrochemicals. Long-lasting chemicals provide a wider time window and decrease the number of applications, which also reduces costs in both the environment and finance.

The chemical synthesis of agrochemicals has to be simple and scalable to allow producing large amounts of chemicals at a low cost. The previous synthesis of tetralone ABA was a six-step linear sequence from 1-tetralone that produced the racemic methyl ester of tetralone ABA with poor overall yields (~15%) [76]. Diddi et al. (2023) reported the two-step synthesis of enantiopure methyl ester of tetralone ABA with high yields (>90%) [91]. Importantly, this simple synthesis scheme can be achieved on an industrial scale. Further chemical and biological progress will be required to collect a series of ABA analogs that are useful in agricultural practices.

## Data Availability

Not applicable.
